# Military Graveyards and Epitaphs in India—IV

**Published:** 1889-03-23

**Authors:** 


### Military Graveyards and Epitaphs in India.—IV.

By an Old Indian.

*(Continued from page 376.)*

The three following, which are selected from a large collection of others of like tenour, though of less point or originality, are good specimens of their fellows, and must, for lack of space, be allowed to speak for themselves. A child encourages its mother in the first to become resigned to her loss on account of the spiritual gain the change has brought with it :—

My mother dear, dry every tear,
And weep no more for me ;
With heart and voice you would rejoice
Could you my glory see.

A soldier, who was probably separated from his family at the time, protests his submission to the call of duty in the second, and thus appeals to the Deity on behalf of these :—

At Duty's call he left his home,
His weeping wife and child,
To gaze on these dear forms again
Heaven that bliss denied.

I go, the spirit groaning cried
While parting from its clay,
Protect, O Lord, my wife and child ;
Thy summons I'll obey.

The third, which is probably taken from some hymn-book, is equally explicit—nay, indeed, more cheering—and the pious sentiment with which it closes is worthy of all acceptation. This is the only place or station in which I met it :

Take this little lamb, said He,
And lay her in My breast ;
Protection she shall find in Me,
In Me be ever blest.
Death may the bonds of life unloose,
But can't dissolve my love ;
Millions of infant souls compose
The family above.

If, now, looking in for a moment at the neat and trim little enclosure at Hoti-Murdan, the head-quarters of the famous "Guides," we will not find much to delay us on our way towards Abbottabad and Serinuggur. Picturesquely situated under the walls of the fort in which the officers and men of that distinguished corps reside, it is only remarkable for the fact that most of those who fell at the Umbeyla Pass are buried in it. I counted upwards of twenty names of men or officers of the 71st Highland Light Infantry, while there were only a few of the 101st, Artillery, or other corps. Hard by is the grave of poor young Ommaney, of the first-named regiment, who, as already indicated, was treacherously assassinated by a Mohammedan fanatic at this place soon, I believe, after the Mutiny ; but the inscription on his tomb merely records this fact, and there is nothing else of any novelty or interest in the place. Leaving then the Peshawur Valley, we come to Attock, but the cemetery here is only noticeable for the beautiful view it commands of the Indus, and as to the square structure at Hussun-Abdul, which is said to contain the remains of the famous Noor Mahal, it is scarcely worth the trouble of a visit.

Struck, as I entered the town of Hummeerpore, on my way towards Huzara, with the appearance of a small pyramidal mortar mound that stands by the wayside, in front of the local gaol and kotwalee (police-station), I dismounted to examine it, and was rewarded for my pains in doing so in this wise:

To the Memory of
Colonel Canara,
Who fell nobly
In the Performance of his duty,
Being summoned
By the rebel Sikh Army
To surrender his guns,
And being basely deserted by his men,
He seized the linstock,
And Fell
Singly combating a host,
July 6th, 1848.

All I could discover about this gentleman's history on the spot amounted to this, that he was an officer of foreign extraction in the old Sikh service, and that he refused to join his corps after the massacre of poor Vans Agnew* by the emissaries of Moolraja at Mooltan, paved the way for the second Sikh war. He paid the penalty of his disinterestedness and loyalty with his life, and assuredly no one could pay dearer for either.

Arriving at Abbotabad† on the 5th of July, 1869, on my way to Cashmere, I visited, as is my wont, the local grave-yard the same evening. I found it "a neat, well-kept little place, which is situated on a slight eminence immediately in rear of the station. It commands a view of the entire valley, as well as, indeed, of the noble hills, including the Sanitorium of Thundiani and the Peak (9,000 feet high) of Meer-Jâni, and the soil on which it stands is dry and sandy." It contained thirteen "pucka" graves on the occasion of my visit, *i.e.*, graves that were surrounded with stones, rails, etc., or that bore memorial crosses. Four others had no such protection or coverings, and some four or five similar receptacles were only distinguishable from the adjacent grass by their elevation. One of these contained, as I was subsequently informed, the remains of a Eurasian band-master, whose acquaintance I had made during the Black Mountain Campaign of the previous year, who had recently committed suicide. The following inscription, which covered the grave of a woman, was the only one of its kind that struck me as being out of the common at this visit—

Life is uncertain,
Death is sure;
Sin the wound, and
Christ the cure.

There was a curious and withal very suggestive monument over the grave of one David Keith, M.D., a medical officer, who died here in July, '53, which had, of course, a special attraction for me. This consisted of a piece of irregular disjointed granite-rack that was placed on a small limestone platform, and contained no other notice than that of the name and date given above. Its central position seemed to imply that Dr. Keith was the first occupant of this spot. Hard by was the grave of a Captain W. W. Repton, who died at the age of 31, which bore an inscription, "Man That Is Born of Woman," etc., from the Prayer-book, and the touching simplicity of the subjoined notification will relieve me from the necessity of criticizing it further. Its pathos is deep enough without being enhanced by utterance of mine:—

Sophia Julia White
Who departed this life on the
30th of June, 1861
Aged 23 years.

The interior of the little church which is situated in the same enclosure, and in which all the local Anglican services are conducted, more than compensated for the lack of memorials noted without. On visiting the interior of this structure the following day, I found that it contained several marble slabs with appropriate inscriptions, the most noticeable of which I will here transcribe. The first on the left, as one walks up the aisle towards the Communion Table, runs as follows:—

To the Memory of
Captain John Paton Davidson
2nd in Command 1st Punjaub Infantry
Who nobly fell
In the defence of his post, The Crag Picket
At the Umbeyla Pass
The 13th of November
1864.

The second is cast in this wise, and needs no further introduction at my hands:—

To the Memory of
James Stuart Oliphant
Who died of wounds received in action
At the Umbeyla Pass
Novr. 24th, 1864
On board The Simla
Off Aden.

But more important than these in so far as that they commemorate the deeds and death of men of greater local prominence or more historic renown are the tablets that are dedicated to the memories of Colonel James and Major Adams. The former—who, I may add, wrote the best Settlement Report I am acquainted with, about the Peshawur district—served for many years on this frontier, and his name is still mentioned with respect by those who knew the value of his tribal knowledge, or could appreciate the extent and disinterestedness of his labours. Though more exposed, inasmuch as he was more isolated than his confrère and successor, he died peaceably in his bed, while the latter fell a victim, as we have already seen, to assassination. Both deserved all the praise their friends or Government bestowed on them.

In Memory of
Hugh Rees James
A Compn. of the Bath and Coms. of the Peshawur Division
Who died at Abbottabad on the
10th of October 1864
Aged 41 years.

A man of great ability, calm and self-reliant in danger, and distinguished for his knowledge of the frontier tribes. His firmness, wisdom, and fortitude are held in honourable remembrance by men of many races. This tablet, erected by his comrades on the frontier, records their admiration of his character.

Equally explicit and discriminating is the inscription that records the murder of poor Major Adams, with this difference, however—that while the record of this event at Peshawur testifies to the fact that he fell at that place on the 22nd January, 1865, that at Abbottabad transfers this sad occurrence to the 23rd of the same month. In other words, there is a difference of a whole day between the two notices. Now, seeing that the latter place is little more than fifty miles—if so much, indeed—as the crow flies, from the former, while both are under the same political régime and postal arrangements, it is not easy to see how such a discrepancy could occur. Yet there it is, as I can testify from careful personal observation, and the occurrence of such an error or misstatement on such an occasion, and under such circumstances as are here set forth, testifies better than any words of mine to the difficulties that antiquarians and others sometimes experience in unravelling disputed points of chronology or date. But we need not dwell further in this place on this feature of the case, and we must leave to others the task of accounting for the discrepancy here referred to.

In Memory of
Robert Roy Adams
Deputy Commissioner in the Punjab
Assassinated at Peshawur on the
23rd of January, 1865.

Here, in Huzara, where, always with wisdom, courage, and consideration, he did his duty to the Government and to the people, it is fit that his name should stand inscribed as a devoted soldier of the frontier.

*(To be continued.)*

* The inscription that was placed by, I believe, order of the Government, over the grave of this unfortunate gentleman and his equally hapless companion, was transferred verbatim by Mr., now Sir Edwin Arnold to his "Dalhousie's Administration of British India," and need not be reproduced here.

† This is the cemetery to which the bodies of the late Major Beatty and Captain Urmston were carried from Oghi. It will also, in all human probability, be the last resting-place of Captain Beley, as well as, indeed, of any other officer who may fall during our operations in the Black Mountain. It is beautifully situated on a hill that overlooks the plain below, and is, I think, the only consecrated grave-yard in Huzara. The soldiers called this "Mary-Jane."

